# An *ex-vivo* model for the biomechanical assessment of cement discoplasty

**DOI:** 10.3389/fbioe.2022.939717

**Published:** 2022-09-02

**Authors:** Salim Ghandour, Konstantinos Pazarlis, Susanne Lewin, Per Isaksson, Peter Försth, Cecilia Persson

**Affiliations:** ^1^ Division of Biomedical Engineering, Department of Materials Science and Engineering, Uppsala University, Uppsala, Sweden; ^2^ Department of Surgical Sciences, Uppsala University Hospital, Uppsala, Sweden; ^3^ Stockholm Spine Center, Stockholm, Sweden; ^4^ Division of Applied Mechanics, Department of Materials Science and Engineering, Uppsala University, Uppsala, Sweden

**Keywords:** spine, mechanical properties, discoplasty, bone cement, disc degeneration, papain, *ex-vivo*

## Abstract

Percutaneous Cement Discoplasty (PCD) is a surgical technique developed to relieve pain in patients with advanced degenerative disc disease characterized by a vacuum phenomenon. It has been hypothesized that injecting bone cement into the disc improves the overall stability of the spinal segment. However, there is limited knowledge on the biomechanics of the spine postoperatively and a lack of models to assess the effect of PCD *ex-vivo*. This study aimed to develop a biomechanical model to study PCD in a repeatable and clinically relevant manner. Eleven ovine functional spinal units were dissected and tested under compression in three conditions: healthy, injured and treated. Injury was induced by a papain buffer and the treatment was conducted using PMMA cement. Each sample was scanned with micro-computed tomography (CT) and segmented for the three conditions. Similar cement volumes (in %) were injected in the ovine samples compared to volumes measured on clinical PCD CT images. Anterior and posterior disc heights decreased on average by 22.5% and 23.9% after injury. After treatment, the anterior and posterior disc height was restored on average to 98.5% and 83.6%, respectively, of their original healthy height. Compression testing showed a similar stiffness behavior between samples in the same group. A decrease of 51.5% in segment stiffness was found after injury, as expected. The following PCD treatment was found to result in a restoration of stiffness—showing only a difference of 5% in comparison to the uninjured state. The developed *ex-vivo* model gave an adequate representation of the clinical vacuum phenomena in terms of volume, and a repeatable mechanical response between samples. Discoplasty treatment was found to give a restoration in stiffness after injury. The data presented confirm the effectiveness of the PCD procedure in terms of restoration of axial stiffness in the spinal segment. The model can be used in the future to test more complex loading scenarios, novel materials, and different surgical techniques.

## Introduction

Approximately 70%–80% of all adults will suffer from back pain at some point in their lifetime (Frymoyer, 1988; Andersson, 1998). Degenerative disc disease is a common source of back pain (Marcolongo et al., 2017; Wilke and Volkheimer, 2018). Depending on the severity, it can also lead to degenerative scoliosis and spinal stenosis (Pritchett and Bortel, 1993; Ploumis et al., 2007). While some patients can be treated by non-surgical means such as physiotherapy, others need surgical intervention to reduce pain and restore functionality for daily living (Rubin, 2007).

Spinal fusion is the gold standard surgical technique for treating degenerative disc disease (Phillips et al., 2013). In the United States, around 455,500 fusion procedures were conducted in 2018, with an increasing procedure count each year (Martin et al., 2019; McDermott and Liang, 2021). It is considered the most costly operating room procedure in the United States totaling $14.1 billion in aggregated costs (McDermott and Liang, 2021). The procedure involves the use of instrumentation such as screws, rods, and cages to fixate the adjacent vertebrae and thus promote bony fusion (Marcolongo et al., 2017). However, for certain patient groups with underlying comorbidities, the risks of the procedure may outweigh the potential benefits (Sola et al., 2018). This is especially the case for elderly patients with advanced degenerative disc disease and associated deformity where the surgical option, a lengthy instrumented fusion, carries a risk of adverse events of 60%—resulting in increased risks for the patient’s well-being (Scheufler et al., 2010; Sola et al., 2018).

**FIGURE 1:**
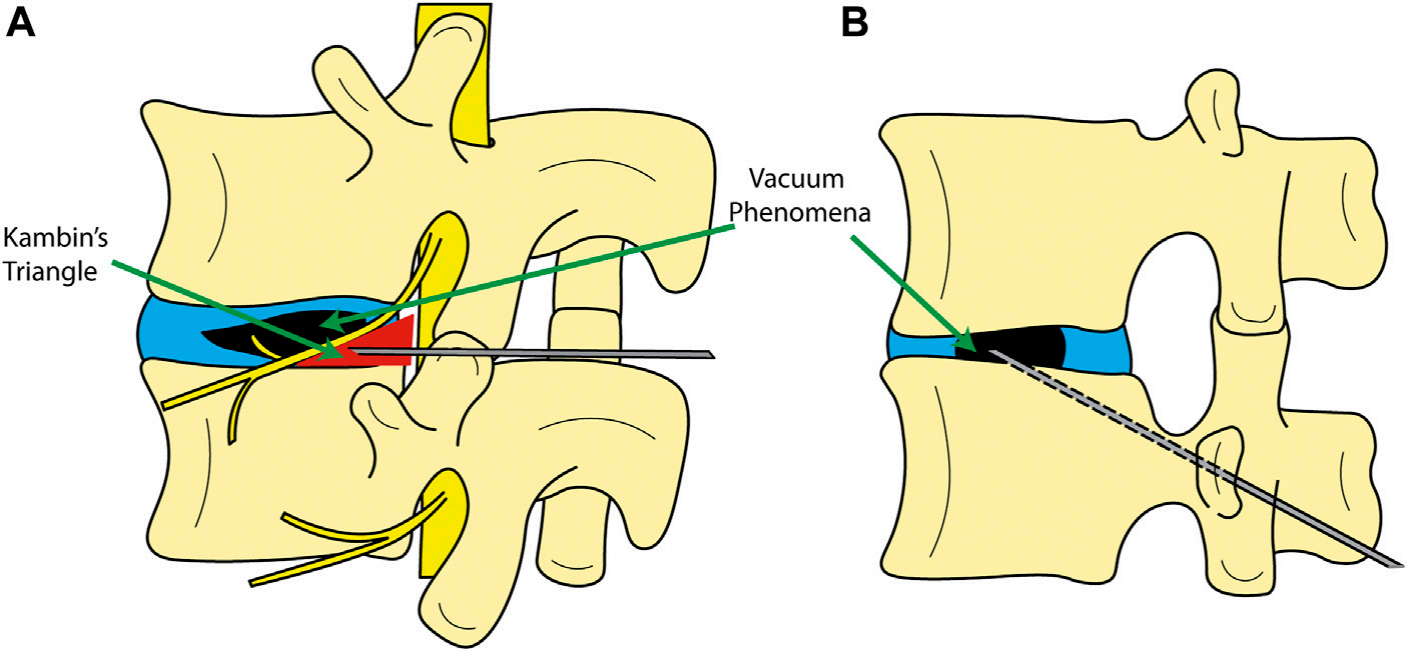
Schematic showing different entry methods to the disc for cement discoplasty. **(A)** entry from Kambin’s triangle; **(B)** transpedicular approach.

For the reasons above, a low-cost, minimally invasive option to spinal fusion has been sought-after for high-risk patients. The use of a minimally invasive procedure typically translates to less blood loss, tissue damage, and risk of infection-related complications and rapid mobilization (Jaikumar et al., 2002; Uddin et al., 2015; Varga et al., 2015). Injection of bone cement into the disc was indeed performed and studied in the last half of the 20th century as a means for low-cost spinal fusion; particularly in the cervical spine (Hamby and Glaser, 1959; Grote et al., 1970; Böker et al., 1989; Van Den Bent et al., 1996). However, it was shown through randomized clinical trials that the use of bone cement in the disc does not yield better fusion results as opposed to keeping the disc space hollow in anterior cervical discectomy with fusion (ACDF). Thus, bone cement for spinal fusion was no longer recommended (Van Den Bent et al., 1996). Recently, Yamada et al. (2016) and Varga et al. (2015) have reported on similar minimally invasive procedures in degenerative deformities, commonly referred to as percutaneous cement discoplasty (PCD) (and in some cases percutaneous intervertebral-vacuum polymethylmethacrylate injection (PIPI) (Yamada et al., 2016; Yamada et al., 2021)). This procedure is based on injecting bone cement into a degenerated disc in the lumbar spine but with a different objective. PCD does not aim to fuse the vertebrae, but rather to treat patients who cannot undergo spinal fusion surgery due to the associated risks of the procedure, with the aim of providing pain relief and improved functionality. PCD can be performed in advanced degeneration of the disc when characterized by a vacuum phenomenon (VP) (Figure 1) (Camino Willhuber et al., 2020a). Vacuum phenomenon is commonly associated with advanced degeneration of the disc and has been reported in 20-35% (Resnick et al., 1981; Morishita et al., 2008) or even as high as 50% (Gohil et al., 2014) of elderly patients, with increasing prevalence with age (Lardé et al., 1982; Gohil et al., 2014). At present, two methods for the introduction of the Jamshadi needle have been developed; either through the Kambin's triangle (Figure 1A), or a transpedicular approach from the lower vertebra (Figure 1B). So far, the cement employed for this procedure is polymethylmethacrylate (PMMA), otherwise used in the spine for vertebroplasty and kyphoplasty, i.e., percutaneous cement injection for stabilization of vertebral fractures. The cement components are mixed in the surgical theatre and injected into the disc with the aid of fluoroscopy. PCD has been shown to provide a consistent pain relief (Camino-Willhuber et al., 2021; Yamada et al., 2021). This pain relief is hypothesized to be the result of increased stability of the spinal segment and partial restoration in disc height after bone cement injection (Kiss et al., 2019). Additionally, PCD allows for a higher degree of motion preservation of the segment compared to spinal fusion, thus there may be a lower risk of accelerated degeneration in adjacent segments (Park et al., 2004).

Since the introduction of PCD, several cohort and case studies have been published on cement injection into the disc in elderly patients with degenerative lumbar scoliosis (DLS). These report significant improvement in patient disability, pain, and quality of life. Yamada et al. reported on mean improvements in Visual Analog Scale (VAS) scores of –55.3 (*n* = 100) for patients who opted for PIPI and –1.9 (*n* = 61) for patients who chose conservative treatments, at 1-month follow-up. Similarly, improvements in American Academy of Orthopedic Surgeons MODEMS version of the Oswestry Disability Index (mODI) (Fairbank and Pynsent, 2000) were –22.7 and –0.6 for PCD and conservative treatments, respectively. These improvements remained consistent at the 2-years follow-up where mean VAS scores were –52.2 (*n* = 91) and –4 (*n* = 53) and mODI were –20.7 and –1 for PIPI and conservative patients, respectively. A few complications (*n* = 3) were reported where slight cement leakage was observed causing pain, however no major complications occurred (Yamada et al., 2016). PCD has shown similar positive results in a case study conducted by Sola et al. (2018). Another study by Camino Willhuber et al. (2020b) with 82 patients (205 levels) presented a 1-year follow-up with significant improvement to the Oswestry Disability Index (ODI) (Fairbank and Pynsent, 2000) from 62 ± 7.12 preoperative to 36.2 ± 15.47 at 1-year post-operation There were no cardiac-, pulmonary- or thromboembolic complications. However, four patients (7%) needed subsequent decompressive surgery within 90 days because of continued or new radicular pain. There was one patient with a deep surgical site infection and one with fracture of an adjacent vertebra that was treated with vertebroplasty. Moreover, after minimum 2 years follow-up, significant and continued improvement to both VAS and ODI scores of approximately 45% and 25% respectively (*n* = 156) was observed (Camino-Willhuber et al., 2021). Yamada et al. (2021) reported on the long-term effects of PIPI in a single center. Both ODI and VAS scores were significantly improved for PIPI patients as opposed to the control group. VAS and ODI improvement for PIPI patients (*n* = 80) at final follow up of 63.7 ± 32.4 months was 72.5% and 57.5% respectively while the control group with non-operative treatment (*n* = 53) had a minor improvement of 5.7% and 17.0% respectively.

While the clinical data seems promising, further investigation into the biomechanics of the treated spinal segments is deemed required to support future advancement of PCD. Techens et al. (2020) outlined the need for a biomechanical model of PCD to study the effects of the procedure on the spine. A porcine model of a degenerated disc construct was developed, using functional spinal units (FSU) comprised of two adjacent vertebra and the intervertebral disc in between. The FSUs were tested with a bending moment of 5.4 Nm using a force offset, and the authors found no significant effect on the mechanical behavior of the segment from treating the injured segments with cement. However, nucleotomy was used to simulate VP, i.e., complete removal of the nucleus pulposus, and no void/vacuum volumes were reported, nor other types of loading. Eltes et al. (2021) developed a volumetric method to study the effectiveness of PCD in terms of decompression, i.e., the volume increase of the neuroforamen that is responsible for relieving the nerves from pressure. It was concluded that after PCD there was an increase in the neuroforaminal canal and a significant positive correlation (*p* = 0.001) between the volume of cement injected and increase in neuroforaminal canal volume. A finite element study exploring different PMMA cements for discoplasty showed a decrease in stresses exerted on the endplates with softer cements (Lewin et al., 2022). The study also advocates for a validation model with consideration to the clinical setting.

The relationship between PCD, the stability of the spinal segment, and disc height differences have not been clearly established yet, therefore the effect of PCD on patients from a biomechanical perspective and whether or not pain relief is a result of that effect is not clarified. Hence, there is a need for a clinically relevant model to investigate new materials and the biomechanical effects of PCD in a controlled setting. The aim of this study was to develop an *ex-vivo* model to enable biomechanical evaluation of PCD in a repeatable and clinically relevant manner. To this end, a papain enzyme buffer was used to produce a repeatable void size in ovine vertebral FSU segments, which were compared to clinical computed tomography (CT) data to ensure clinical relevance. The FSUs were mechanically tested under compression before and after injury, as well as after PCD to measure stiffness, which directly correlates to the stability of the segment. It was hypothesized that a clinically relevant injury with vacuum voids would decrease the stiffness of the vertebral segments, and that the stiffness would be increased after discoplasty. This model could enable a deeper understanding of the biomechanics of discoplasty and serve as a basis for further investigation of the procedure, including evaluation of new biomaterials.

## Materials and methods

**FIGURE 2:**
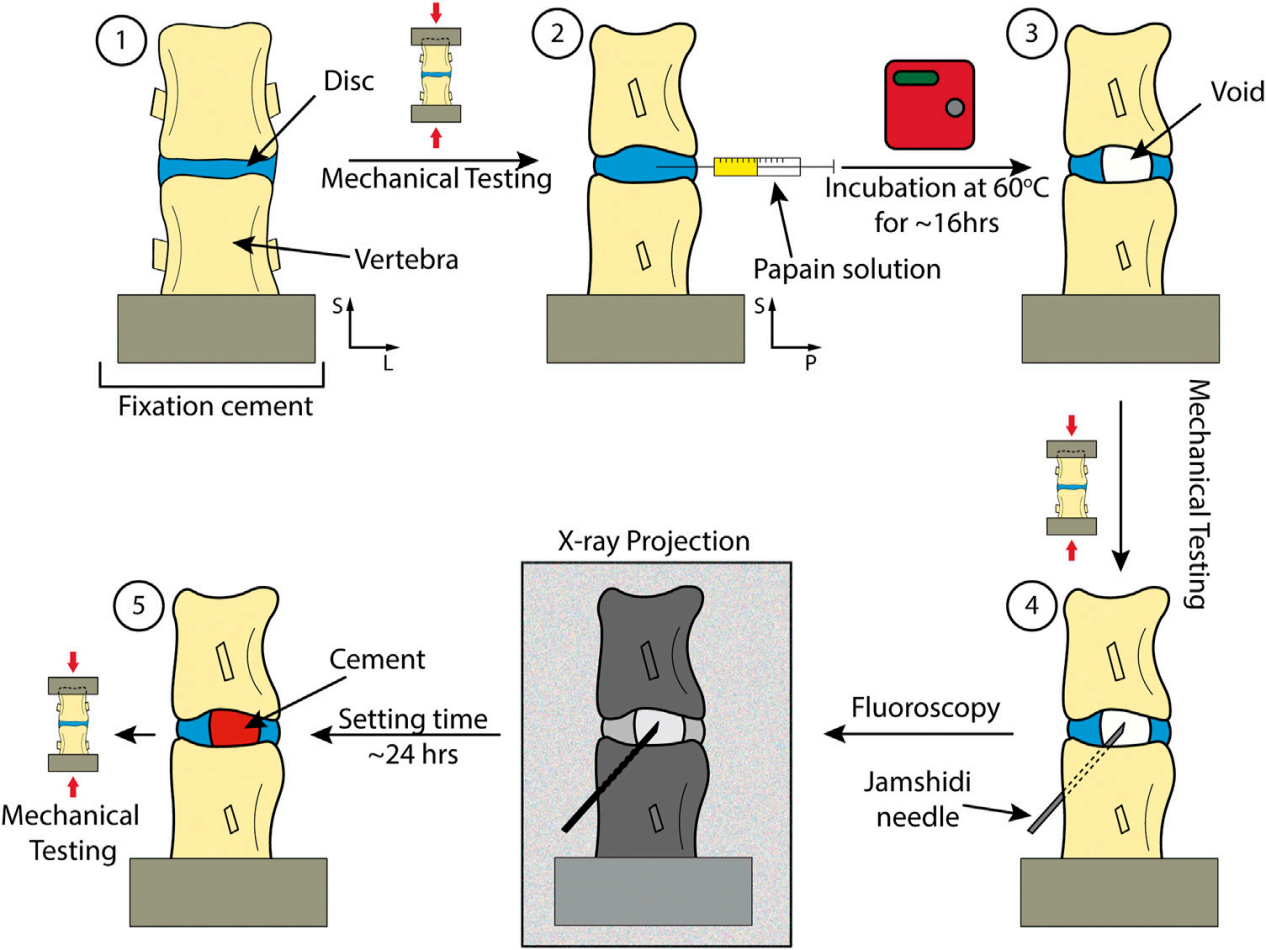
Key steps in the study including different checkpoints for mechanical testing. 1) Dissection and preparation of the spinal segment. 2) Injection of the papain. 3) Incubation of the sample to lead to injured disc. 4) Performing discoplasty on sample. 5) A treated sample with PMMA.

A summary of the procedure can be found in Figure 2. In brief, FSUs were dissected from fresh ovine spines. They were then submerged in PBS +1%vol pen-strep for 12 h before each step in the procedure. Each FSUs was mechanically tested non-destructively as harvested (healthy), after papain induced VP (injured), and after discoplasty treatment (treated). The samples were scanned with micro-CT before and after tests to ensure no macro fractures were present in the adjacent bone. The VP was produced using a papain solution that was injected into the disc. Micro-CT images were used to assess void and cement volume as well as disc morphology. The segments were then treated using PCD. The following sections present the different steps in more detail.

### Sample preparation

**FIGURE 3:**
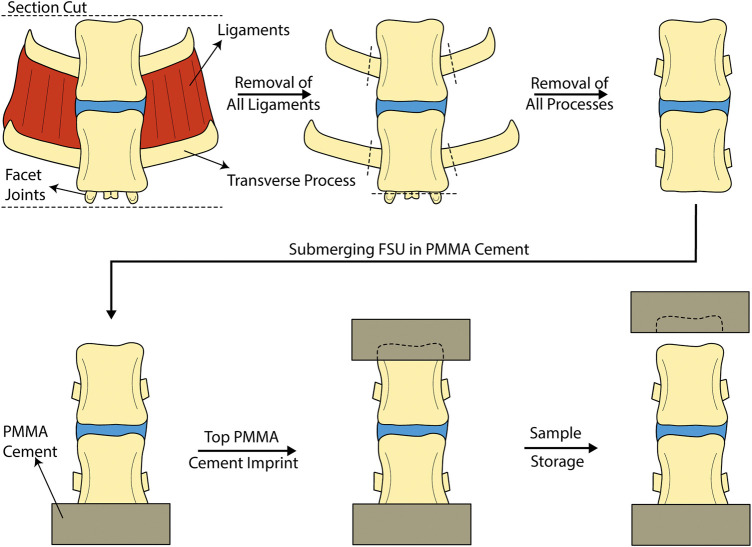
Procedure for the dissection and preparation of ovine FSUs for mechanical testing.

The spines originated from Leicester crossbreed female sheep (adult sheep above the age of 2.5 years) from the Uppland region in Sweden. It has previously been shown that ovine spine possess similar biomechanical properties as human spine in terms of range of motion (Wilke et al., 1997a) and are commonly used in biomechanical evaluations of spinal treatments (Wilke et al., 1997a; Wilke et al., 1997b; Reid et al., 2002). The spines were harvested and dissected fresh in the lab where they were rinsed and divided into individual FSUs. Clinically, PCD has been performed on the lower thoracic and lumbar spinal segments, thus similar segments were considered for the experiments (T11-L6). All spinal processes were cut off to ensure the forces were transmitted purely in the disc during mechanical testing. The purpose of the mechanical testing was to test the performance of the material in the disc, preserving other ligaments and joints that exert forces may mask issues related with the implant (Berger-Roscher et al., 2017). This also facilitated micro-CT analysis. The caudal vertebra was submerged in PMMA, and an impression of the top endplate was created using a separate PMMA mould. The segments were then stored in −20°C for later use. A total of 12 FSUs were dissected and used for this study. This process is outlined in Figure 3.

### Injury method

Voids were induced in the disc using a 60 U/ml papain buffer containing 55 mM sodium citrate, 150 mM sodium chloride, 5 mM ethylenediaminetetraacetic acid (EDTA), and 77 mM cysteine-hydrochloride (all purchased from Sigma-Aldrich, Darmstadt, Germany). Papain buffers have previously been shown to induce disc degeneration by digesting disc tissue (Roberts et al., 2008; Chan et al., 2013; Malonzo et al., 2015). Preliminary tests were conducted to establish the protocol for injection and inspection of the samples. Doses of 50 μl were injected to control the damage and monitor the VP evolution. The FSU was scanned in a Skyscan 1172 micro-CT (Bruker Corporation, Massachusetts, United States) to measure the distance between the anterior and center of the disc. The distance was recorded and marked on the needle to ensure proper injection from the anterior of the sample. After injection, the FSU was subsequently incubated in humid conditions at 60°C for 16 h to allow the papain to digest the disc (Malonzo et al., 2015). The FSU was scanned again, and the void size and morphology was assessed. Reinjection was required under two conditions: if the void size was too small to be injected with cement or if the void did not extend to one (type 2A) or both (type 3A) endplates (Camino Willhuber et al., 2020a). The sample was discarded if the papain injection had created a hole in the annulus visible by eye or micro-CT.

### Imaging

Screening, fracture assessment, injury morphology, and cement morphology assessments were conducted using micro-CT reconstructions. For screening and preliminary macro fracture assessment visual inspection of the images was conducted. The total time for each scan was approximately 20 min. A voxel size of 27.16 µm was used to have maximum field of view. The source voltage and current were set at 100 kV and 100 µA respectively and the samples were exposed for 1600 ms per image. Two frame averages were used to reduce some noise, a rotation step of 1°, and 180° rotation to keep scanning time to a minimum. It was crucial to keep the scanning time low in order not to lose any information after compression of the samples. An Aluminum + Copper filter was used as it captured both soft tissue and hard tissue with good detail. All samples were scanned with the same settings to ensure an unbiased comparison in segmentation.

Injury and cement morphology were analyzed using open-source software, 3D Slicer (Fedorov et al., 2012), where image segmentation of the void, disc and cement injected was conducted. Void and disc tissue were segmented using “growth from seed” method which identified changes in average voxel greyscale from markers that were manually input in the system. Using an iterative method of marking for each individual scan, the volumes were established. A similar procedure was performed for the clinical CT images provided. Anonymized CT images of spines pre- and post-operation were provided by a collaborating hospital for 3 patients. As the method is semi-automatic it was verified by an independent researcher. Three random void volume percentages were assessed by this independent researcher and compared with the results from the study, giving a margin of error of approximately 1%. For the cement volumes, the segmentation was done using an automatic threshold method as it could be differentiated using the histogram (Otsu, 1979).

**FIGURE 4:**
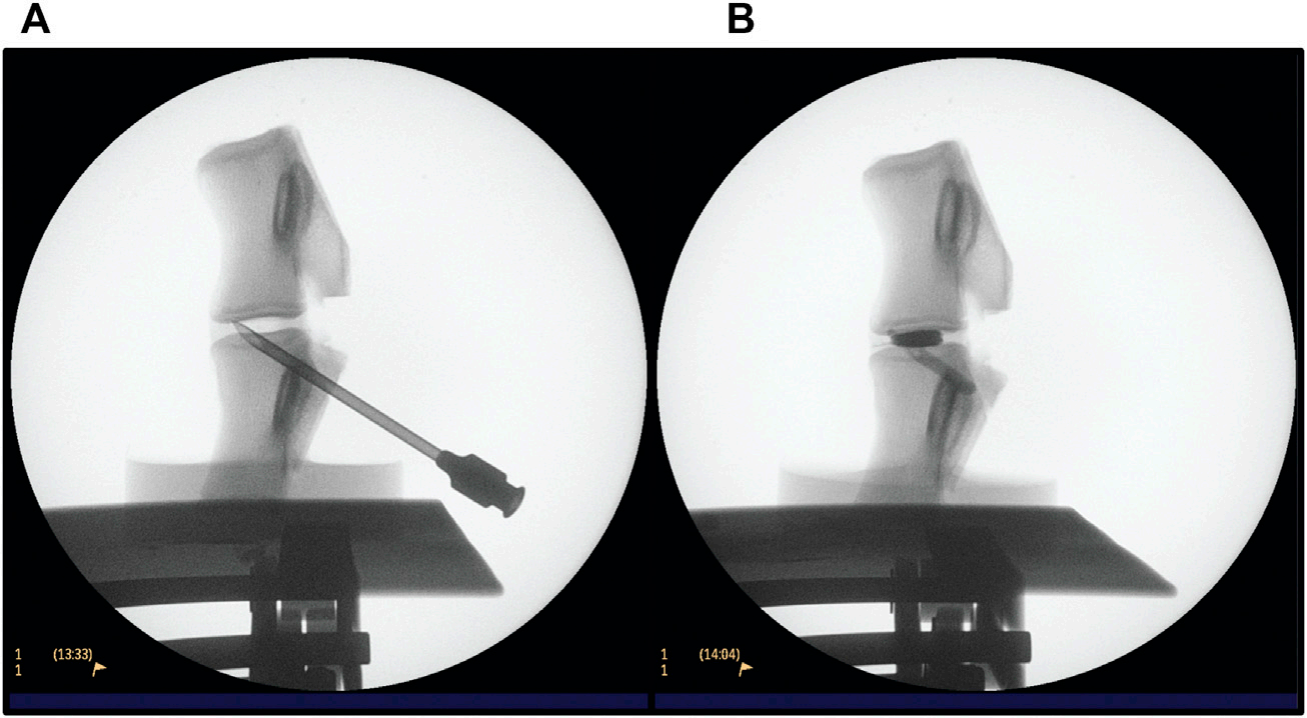
Fluoroscopy procedure for conducting PCD on an L3-L4 FSU. **(A)** verifying the position of the needle **(B)** after injection of PMMA cement.

Fluoroscopy was used while performing the PCD. A Philips BV Endura C-arm was operated by the surgeon (KP) and used to guide the needle into the disc and monitor cement injection (Figure 4). ‘Head/Back’ optimized settings were used on the machine, 45 kV and a range of 0.198–0.475 mA was used. Exposure time was kept to a minimum. On average, each sample was exposed to a dose of 1 µGy.

### Cement discoplasty procedure

The FSUs were handled by the surgeon carrying out the discoplasty. The transpedicular approach was favoured to prevent physical damage to the annulus fibrosis—highlighting the advantage of using papain injection instead of nucleoplasty. Further, it reflects the protocol that is conducted at the collaborating hospital. As sheep have a higher bone density, a tabletop drill was employed to create a hole from the posterior pedicles into the void. Once an appropriate hole was drilled, it was verified using fluoroscopy and the needle was placed using light tapping (Figure 4A). When the position of the needle was considered appropriate, radio opaque liquid (Omnipaque, GE Healthcare, United States) was injected to confirm the void morphology. The liquid was then retracted, and PMMA cement V-steady (G21 s.r.l, San Possidonio, Italy) was mixed according to manufacturer’s instructions and injected until the void was filled (Figure 4B). This is a cement designed for vertebral body augmentation, containing e.g., a higher amount of radiopacifier for enhanced visibility, which is typically the type of PMMA used for PCD (Sola et al., 2018; Camino Willhuber et al., 2021). Finally, the needle was retracted while some cement was injected to fill up the space displaced by the needle.

### Mechanical testing

**FIGURE 5:**
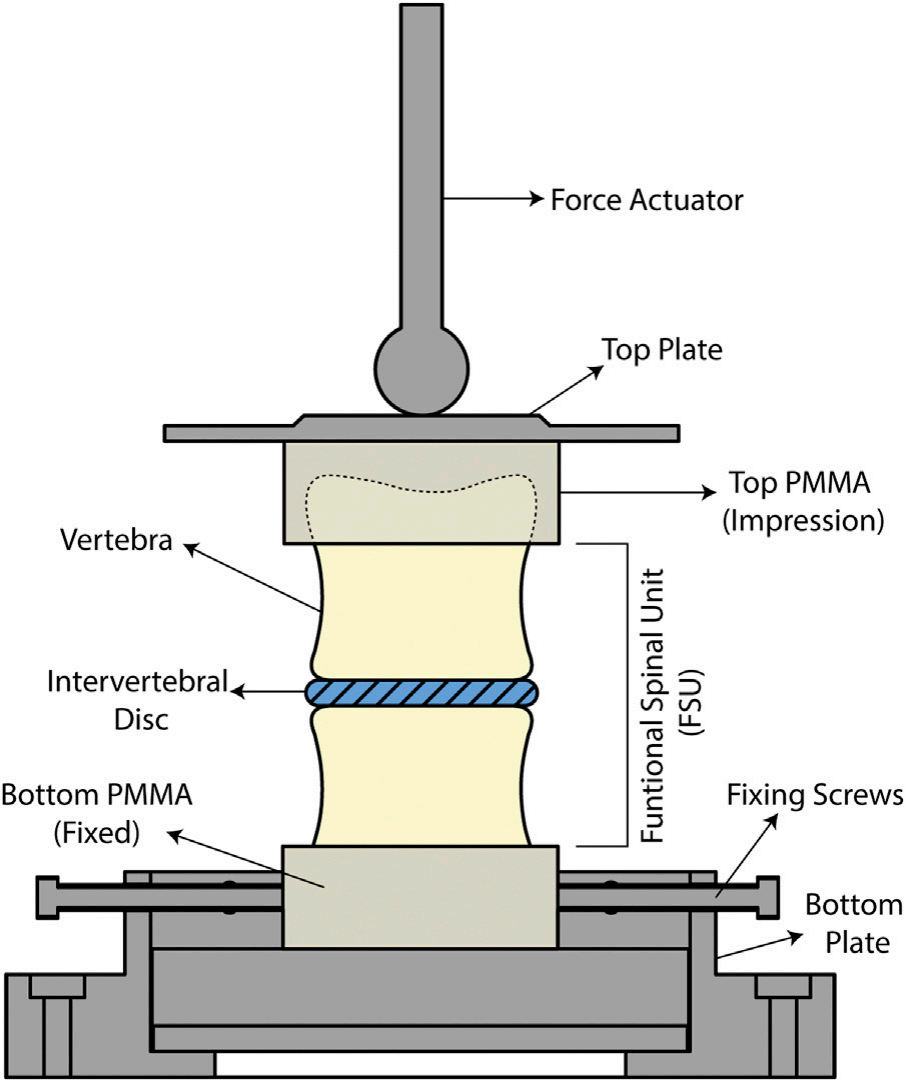
Schematic of the mechanical testing setup.

Compression testing was done using an MTS 858 Mini Bionix T/II (MTS Systems Corporation, Minnesota, United States) to assess the stiffness of the FSUs. The stiffness of the FSU in compression directly correlates to the stability of the segment. A displacement rate of 5 mm/min, or an average strain rate of 0.017%/s, was used and a maximum displacement of one third of the minimum disc height was chosen. This was calculated by finding the minimum distance parallel to the vertical direction between the vertebra using reconstructed micro-CT scans. Previously used strain rates for testing human intervertebral disc tissue are in the range of 0.01–0.8%/s (Newell et al., 2017), and a strain rate in the lower end was chosen to capture sufficient data for estimating stiffness. The displacement limit was determined by pilot studies and allowed for standardization of the displacement cycles as each sample had a unique geometry. A preload of 25 N was set and five compression cycles were performed per sample where the last three cycles were used for stiffness measurements. Initial investigations showed that 25 N was the minimum force where the entire assembly is in contact (i.e., the superior vertebra is conformed to the impression on the PMMA), and three cycles were sufficient for preconditioning the samples and the last three cycles were nearly identical (shown in Supplementary Material S1). The setup featured a ball on plate setup to ensure the actuated force is exerted in the center, vertically at a single point, and reduce bending moments as the top plate pivots freely (Figure 5). The bottom PMMA holder was fixed using screws and the sample was positioned so that the center of the top endplate was aligned with the actuator. The stiffness of each segment was estimated in the linear range, corresponding also to human disc axial strains during physiological loading (2%–5%) (Tsantrizos et al., 2005; Tavana et al., 2021).

### Statistical analysis

Statistical analysis was done using SPSS Statistics Version 28 (IBM Corp., Armonk, NY, United States). Sample size was calculated using G*Power 3.1.9.7 (Erdfelder et al., 2009). Since there were no previous studies with a similar method, estimating the effect size for clinical relevance proved to be difficult. The sample size was calculated after the experiment was conducted to ensure conclusive results and significance of the statistics. The number of samples calculated was 6 from both the stiffness’ and disc height’s effect size, given by a partial eta squared $\left(\eta_{p}^{2}\right)$ of 0.576 and a power of 95%. The partial eta squared was calculated using multivariance analysis on SPSS based on the means of the three health groups. A repeated measure analysis of variance (ANOVA) was used, as the same specimens were used in each group. Normality of the data was established using Shapiro-Wilk’s normality test. All groups showed significance above 0.05. Further, the repeated-measures ANOVA was validated using Mauchly’s sphericity test. For testing correlations, Pearson’s correlation coefficient was used, and a two-tailed test was employed to test for significance. Significance was considered for *p* < 0.05.

## Results

### Void and cement volumes

**FIGURE 6:**
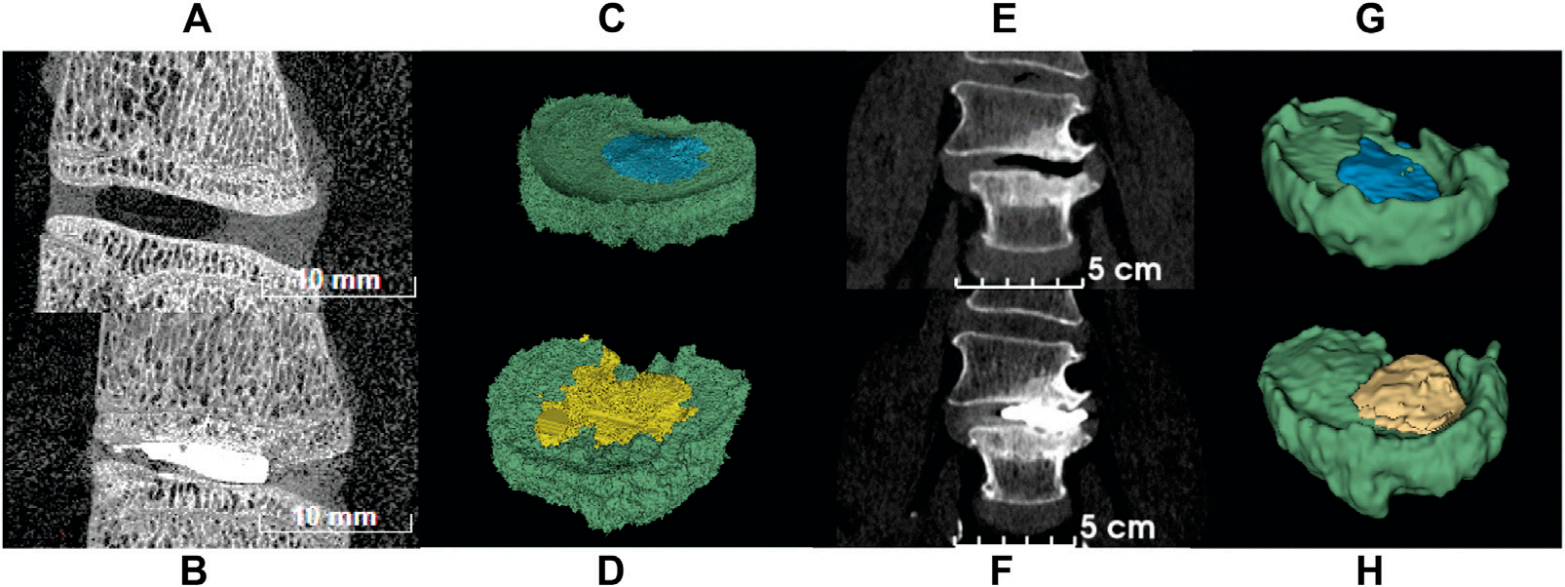
CT images of ovine and human spine segments before (top row) and after (bottom row) PCD. **(A)** Sagittal view of ovine FSU with papain induced void; **(B)** Sagittal view of ovine FSU after PCD; **(C)** Segmentation of ovine FSU featuring void and disc tissue; **(D)** Segmentation of ovine FSU featuring cement injected and disc tissue; **(E)** Frontal view of human spine with degenerated disc; **(F)** Frontal view of post-op image of human spine after PCD surgery; **(G)** Segmentation of human spine featuring void and disc tissue; **(H)** Segmentation of human spine featuring cement injected and disc tissue. Green = disc tissue; blue = void; yellow = PMMA cement.

The segmentation results showed that ovine samples had 15.4% ± 7.4% void volume and 15.5% ± 5% cement volume (*n* = 11) and clinical data showed 6.6% ± 2.2% (*n* = 3) void and 12.4% ± 4.4% cement volume (*n* = 6). Three of the voids could not be measured accurately due to excessive compression, i.e., not enough slices were available to give an accurate volumetric representation. An example of the segmentation is found in Figure 6. The region of cement analyzed was the cement found inside the disc and does not include cement residue or cement found in the vertebral body.

**FIGURE 7:**
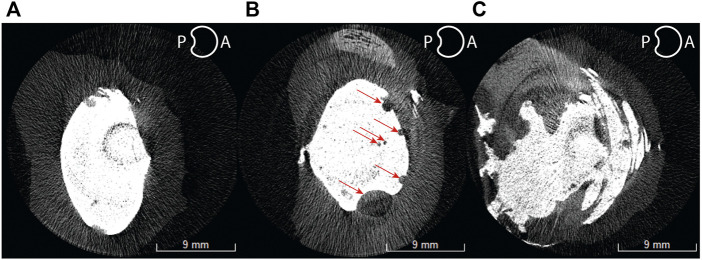
Axial cross-sectional micro-CT slices of a well-filled void **(A)**, under-filled **(B)**, and over-filled **(C)** ovine spine discs. Red arrows indicate bubbles of air trapped in the cement.

Eleven out of twelve samples successfully passed inspection after inducing VP. The rejected sample showed penetration through the annulus and separation of the endplate from the vertebral body due to weakening in the growth plate from excessive papain digestion. A total of approximately 100–150 µL of papain buffer solution was injected into each disc. Variations were found between ovine FSU samples (Table 1). However, all samples had type 2A (*n* = 1) and type 3A (*n* = 10) vacuum sizes as prescribed by a vacuum classification study conducted by Willhuber et al. (Camino Willhuber et al., 2020a). Eleven out of eleven samples successfully passed inspection after PCD. No cement leakage was observed through the annulus. Six samples had the void filled (Figure 7A) while 3 samples had more cement volume than void. This may be due to delamination of the annulus and high injection pressure (Figure 7C). Two samples had less cement volume than void volume due to large air bubbles. This could not be spotted while using fluoroscopy and thus they were somewhat underfilled (Figure 7B).

**TABLE 1:** Volume Percentage of void and PMMA relative to the volume of the disc. Vacuum classification from Camino Willhuber et al. (2020a).

Sample	Void (% of disc)	Vacuum classification	Cement (% of disc)
Sh1 L1-L2	12.3	3A	14.48
Sh1 L3-L4	3.56	2A	9.43
Sh1 T12-T13	20.17	3A	20.38
Sh2 L3-L4	15.6	3A	16.09
Sh2 L5-L6	13.37	3A	11.1
Sh2 T12-T13	12.68	3A	13.86
Sh3 L1-L2	19.4	3A	16.3
Sh3 L3-L4	23.54	3A	24.2
Sh3 L5-L6	30.72	3A	20.4
Sh3 T11-T12	10.27	3A	16.2

### Disc height measurements

**FIGURE 8:**
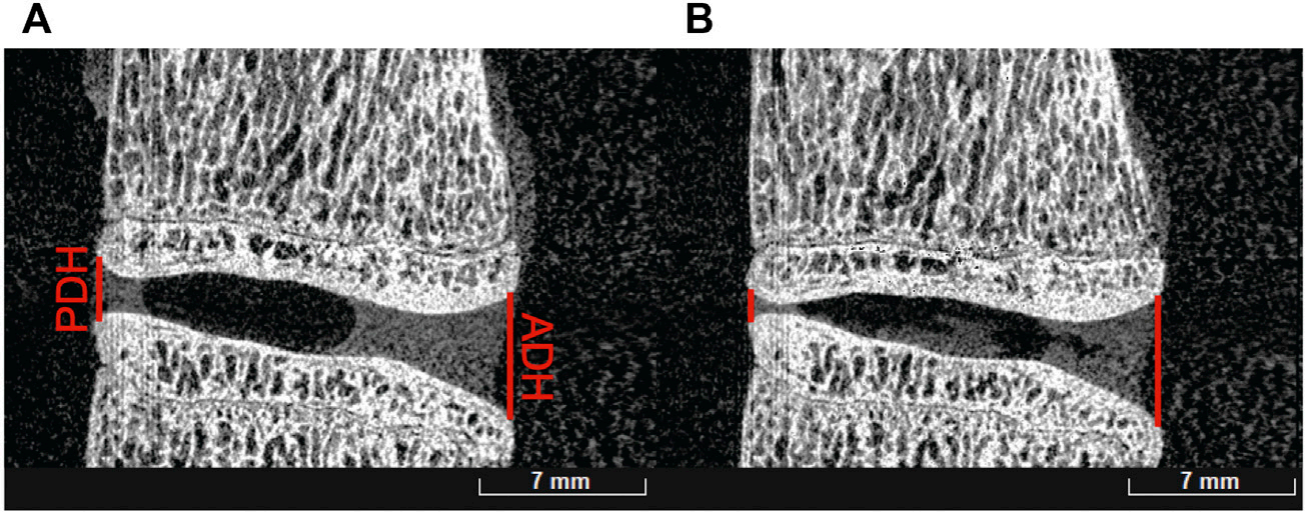
Before **(A)** and after **(B)** mechanical testing of an L1-L2 ovine sample showing posterior disc height (PDH) and anterior disc height (ADH) differences.

All samples were scanned pre and post mechanical testing. An example of the difference of disc height between a tested and untested sample is shown in Figure 8. Due to this difference, all disc height measurements presented are post mechanical testing to simulate muscle and tendon load. The percentage differences were normalized to the healthy disc height for each sample.

**FIGURE 9:**
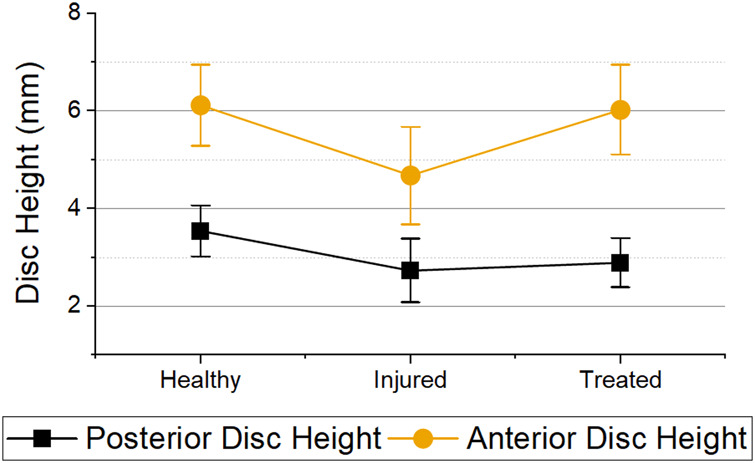
Posterior and anterior disc heights for the different health groups immediately after mechanical testing. Error bars = 95% Confidence Interval. Statistical differences was observed in both anterior (*p* < 0.001) and posterior disc height (*p* = 0.004) between health groups.

A significant difference was observed in the evolution of the anterior (*p* < 0.001) and posterior (*p* = 0.004) disc heights. It was measured that on average, both posterior and anterior disc height after injury were reduced to 76.1% (decrease of 23.9%) and 77.5% (decrease of 22.5%) respectively (Figure 9). In addition, the anterior disc height on treated samples was restored to 98.5% (increase of 22.4%) while the posterior height regained to only 83.6% (increase of 6.1%) of its healthy disc height.

### Mechanical testing

**FIGURE 10:**
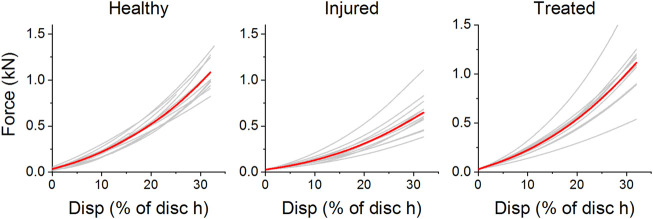
Force-displacement curves of ovine segments as healthy, treated, and injured. The displacement is given as a percentage of minimum disc height for each sample. Curves in red represent the average cycle for each health group.

Eleven out of eleven samples passed all three mechanical tests. Force-displacement curves for each individual sample can be found in the Supplementary Material S1. Figure 10 shows the force displacement curves of each sample in the different groups. All curves showed an increase of stiffness with strain. This trend is similar for all investigated specimens. The increase in stiffness is a result of complex stress stiffening of the discs predominantly at higher levels of compression. It can be noted that the injured segments had a lower stiffness on average than the healthy segments throughout the entire cycle. Furthermore, most treated samples (*n* = 9) showed a similar response to the healthy segments.

**FIGURE 11:**
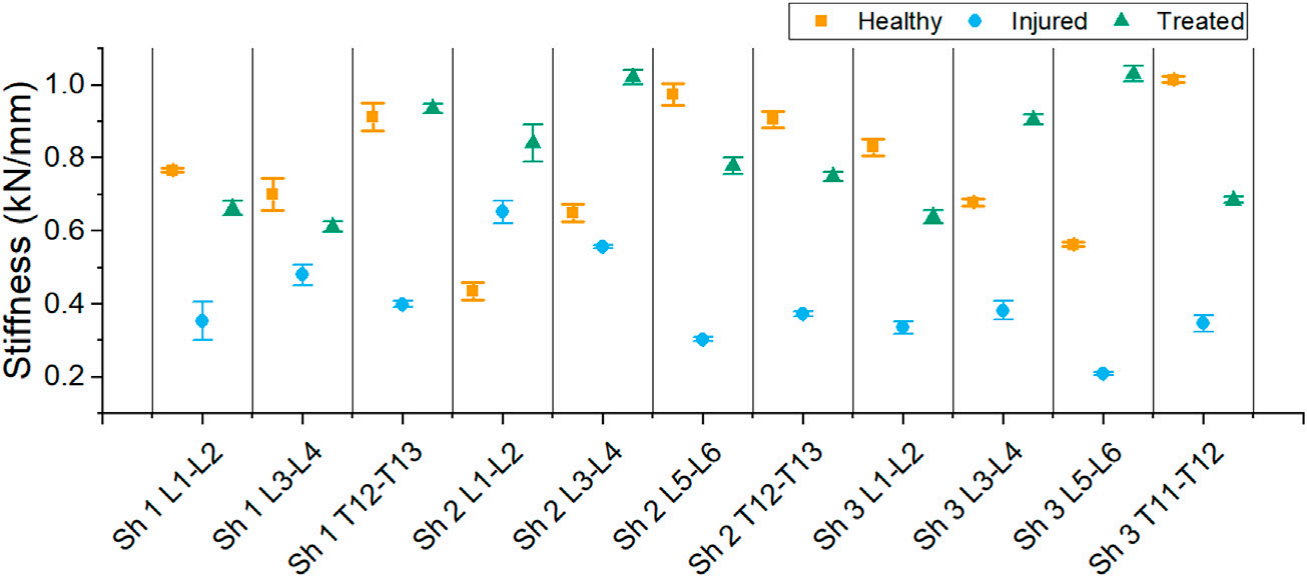
Stiffness of each segment for the different health group: healthy, injured and treated with cement.

The stiffness derived from the linear regression of each sample is reported in Figure 11.

**FIGURE 12:**
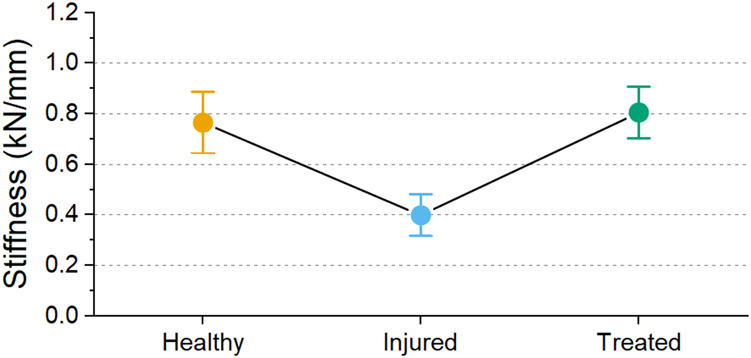
Mean stiffness values for the different groups with 95% confidence interval. Significant difference was observed between health groups (*p* < 0.001).

A decrease in stiffness after injury was found for each sample except one (L1-L2 FSU from sheep 2). However, all treated segments had a higher stiffness than the injured segments. The average stiffness for each group is shown in Figure 12. The average stiffness for the healthy discs was 0.767 ± 0.271 kN/mm. A decrease of stiffness was observed to 0.400 ± 0.184 kN/mm in the injured discs. After treatment, the stiffness of the discs increased to 0.806 ± 0.228 kN/mm.

**FIGURE 13:**
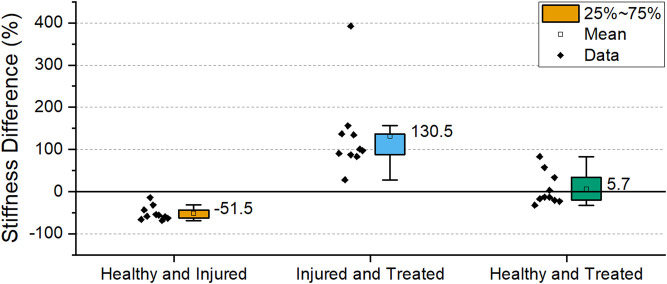
Percentage difference in stiffness of the different health groups. The box indicates the values between 25-75% and the label indicates mean value. (Sh2 L1-L2 excluded).

The stiffnesses differences between the groups was statistically significant (*p* < 0.001). To emphasize the differences, a comparison between the different groups of the samples is shown in Figure 13. Between healthy and injured groups, the stiffness decreased by 51.5% on average while a significant restoration of stiffness is shown when comparing injured and treated groups. Healthy and treated groups show only a difference of 5% stiffness confirming the restoration stiffness observed in Figure 12.

**FIGURE 14:**
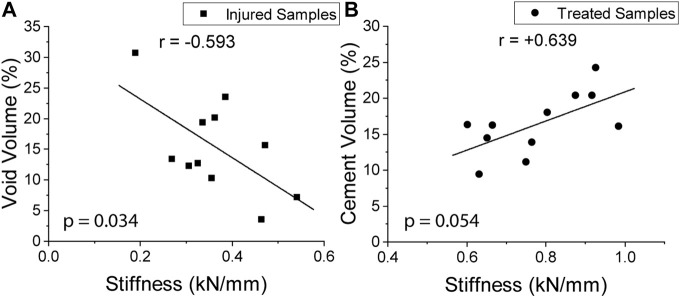
Correlation between stiffness vs. void volume (*p* = 0.034) **(A)** and cement injected (*p* = 0.054) **(B)**.

Excluding the outlier in the study, all samples had a decrease in stiffness between healthy and injured. A statistically significant negative correlation (*p* = 0.034) was observed between void volume size and stiffness (Figure 14A). Likewise, all samples had a stiffness increase as a result of cement discoplasty. However, a statistically significant correlation could not be confirmed between cement volume injected and stiffness at the chosen significance level (*p* = 0.054) (Figure 14B).

## Discussion

The objective of cement discoplasty is twofold. Firstly, to relief patients from LBP caused by the narrowing of the neuroforamen space and decrease in disc height (Varga et al., 2015) or endplate lesions (Yamada et al., 2016). Secondly, to provide stability to the spinal segment (Varga et al., 2015; Sola et al., 2018; Camino Willhuber et al., 2020b). This study presented a procedure to induce a repeatable injury resulting in the clinically observed vacuum phenomenon, and reported on the mechanical properties of healthy, injured and PCD treated ovine functional spinal units. X-ray imaging techniques were employed to non-destructively assess void volume, cement volume, posterior and anterior disc height.

Choosing an appropriate animal model for testing discoplasty was an essential first step in developing this method. Depending on the objective, different animal models have been employed, where the most common are ovine, rabbit, bovine and porcine (Reitmaier et al., 2017). To our knowledge, the only other study on the biomechanical properties of discoplasty used a porcine model (Techens et al., 2020). However, an ovine model was chosen for this study for several reasons. It possesses similar gross anatomical and biomechanical representation to human lumbar spine (Wilke et al., 1997a, 1997b; Reid et al., 2002), a small form factor, and was more accessible in this study relative to porcine. Further, ovine intervertebral discs have been shown to have similar water content in the discs relative to human spine, which directly affects the mechanical properties of the disc (Reid et al., 2002). As long as the loads and implant (i.e., the bone cement volume) are scaled down, a study on ovine samples can be assumed to give a good approximation to how discoplasty would affect human discs. The smaller form factor enabled the use of equipment such as the employed micro-CT, which could not be used with a larger FSU such as porcine.

A vacuum phenomenon needs to be present in order to apply PCD. For the objective of this study, producing a repeatable and clinically appropriate void size in the disc was necessary, but the bone density and quality was not considered as relevant, thus an animal model was used. One of the important advantages of using papain was inducing minimal penetration damage at the point of injection. Therefore, the present void model can be considered more similar to the clinical vacuum situation as opposed to performing a nucleoplasty, which was done in the only previously reported model of discoplasty (Techens et al., 2020). Additionally, the location of the void can be controlled by the injection site. Using CT images as a guide, markers could be set, and injection depth recorded making the procedure repeatable. Over 90% of samples passed the exclusion criteria and all voids were type 2A and 3A, which are within the recommended void type for discoplasty treatment (Camino Willhuber et al., 2020a) making the model suitable for clinical comparison.

The stiffness of the healthy ovine spine could not be directly validated due to the lack of literature on ovine FSUs without facet joints and ligaments. The stiffness decrease after injury was expected, as a result of the digestion of the nucleus pulposus. The reduction of hydrostatic pressure in the disc results in segment instability and a reduced capacity to carry load. Moreover, Chan et al. (2013) have shown that papain also influences the annulus fibrosis fiber composition by observing a decrease of rotational stiffness in their samples.

The stiffness of the treated samples was similar to the healthy samples on average (Figures 12, 13), although variations were found within a sample (Figure 11). The mechanical properties of disc tissue and PMMA are very different, however the behaviour of the FSUs in the 2%–5% range was similar (Figure 10). In both cases, it can be assumed that the majority of the displacement was occurring inside the disc space as cortical bone (E = 15–25 GPa) (Grant et al., 2014) is much stiffer than PMMA (E = 1.5–3.7 GPa) (Kurtz et al., 2005; Boger et al., 2008; Hernandez et al., 2008) and disc tissue (Casaroli et al., 2017). It is worth noting that in most cases, the bone cement was in contact with both endplates thus carrying most of the load in the treated segments. This was supported by the fact that the FSUs showed an increase in stiffness regardless of the amount of cement injected (Figure 11). Overfilling and underfilling of voids has not been studied in a clinical setting as clinical CT scanner resolution is limited. It may be the case that this occurs also in a clinical setting but it is not observed.

To the authors’ knowledge, only one other *ex vivo* study has reported on the biomechanics of cement discoplasty (Techens et al., 2020). The study presents different loading cases such as moment bending but no compression testing. Therefore, the findings from both this study and Techens et al. could complement each other to portray the overall performance of cement discoplasty. Techens et al. (2020) found no statistically significant differences in rotational stiffness in flexion-extension and lateral bending between healthy, injured and treated groups. From a clinical point of view, this is promising, since it would indicate that patients maintain their range of motion of the treated segment while restoring stiffness in compression.

Disc height restoration is also an important outcome from PCD. The procedure is hypothesized to remove pressure on the spinal nerves by increasing the disc height (Kiss et al., 2019). From a clinical point of view, this raised concerns regarding how to expand the disc space during injection, and Varga et al. (2015) recommends using a high viscosity cement. In the present study, a cement described as high-viscosity cement was used, and while anterior disc height was fully restored, the posterior was not (Figure 7). Nevertheless, an increase of 22.4% in the anterior disc height and 6% in the posterior disc height directly increases the intervertebral foramen, which should result in reduced pressure on the spinal nerves. Eltes et al. (2021) drew similar conclusions when measuring the neuroforamen volume in pre- and postoperative CT scans. Similarly, Techens et al. (2020) observed similar disc height trends to this study in their porcine nucleoplasty model—confirming the effectiveness of PCD in that regard. With the present model, different cements with various viscosities and material properties can be tested to assess disc height restoration and validate simulation studies.

Segmentation of the samples was key to compare with clinical data and further the understanding of discoplasty. Techens et al. (2020) compared the volume of cement injected between patients and their samples however no attempt in measuring correlation was performed. Absolute volumes could not be compared as a different animal model was used in this study. Moreover, due to natural variation of animal models the percentage cemented may be a more adequate measure to compare with clinical data. This was addressed herein by the segmentation of micro-CT scans and clinical CT data provided by the hospital. It was shown that similar percentages of cement were injected in both ovine samples and patients. The void volumes were however not similar, which could be explained by the difference in loading scenario in a patient compared to our model. Moreover, the resolution of the clinical CT scans is limited.

One of the limitations of this study was the use of an animal model. While being more accessible, ovine spine has a higher bone mineral density compared to humans. Additionally, all ligaments and processes were removed, altering the biomechanics of the FSUs. Therefore, adjacent vertebral fracture risks in relation to loads experienced and materials used were difficult to assess using this model. Hence, in the case of transferring this model to a human cadaver case retaining ligaments and processes should be considered. Further, due to the time constraint between individual microCT scans, the noise and artefacts produced could not be eliminated for all samples. Another disadvantage was that measuring the exact volume of papain injected into the disc was not possible due to the pressure in the disc. It is recommended that the papain is administered through more than one dose to possess better control over the VP location and size. Delamination of the annulus tissue observed in this study (Figure 7C) was not an expected outcome from the papain injection. Although it did not affect the results, weaker annulus tissue such as from elderly human cadaver FSUs may be affected more severely than healthy ovine FSUs, and therefore papain dose must be minimized. It was observed that injection of papain as the needle was retracted promoted delamination. It is important to note that applying this method on other cadaver models requires some preliminary investigation to measure the volume required per dose and location of injection.

In summary, the method outlined in this study could be used to explore different aspects of discoplasty. Material optimization for the technique is a topic for further investigation. As previously discussed, there is a risk of vertebral facture in PCD (Camino Willhuber et al., 2020b). PMMA has the advantage of conforming to the geometry of the void. Therefore, if the PMMA is conformed to the endplates, there is less risk of concentrated stresses and movement of the PMMA. Osteoporotic patients possess a higher risk for vertebral fractures and have been excluded from some studies (Yamada et al., 2021) or advised to be treated pre-operatively to reduce the risk (Sola et al., 2018). Optimizing the modulus of PMMA could prove to be useful in this case. Low-modulus PMMA was previously proposed for spinal applications (López et al., 2011, 2014; Holub et al., 2015; Persson et al., 2015) and could be beneficial in PCD, particularly for osteoporotic patients. This method could also serve as a screening phase where different surgical advancements can be evaluated without putting patients at risk. The two entry methods could be compared to further the understanding of PCD. Further, different loading scenarios and multiple level discoplasty could also be tested using this method.

## Conclusion

In this study, a methodology to evaluate discoplasty in an *ex vivo* ovine model was established. Papain was used to induce clinically relevant voids, as established by percentage void volume of the disc in comparison to clinical data. The injury significantly decreased the stiffness of the FSUs, as expected. An increase in stiffness was found for the specimens treated with PCD as compared to the injured ones. Injected cement volumes were found to be comparable to those of clinical treatments. The anterior disc height was restored to its healthy state while the posterior disc height was not fully restored after treatment. Compression tests indicated support for PCD as an alternative procedure to fusion for patients with painful disc degeneration with vacuum phenomena, as based on the stiffness and disc height restoration possibilities.

## Data Availability

Ovine microCT images, reconstructions, and mechanical testing data is available *via* Zenodo using the following DOI: 10.5281/zenodo.6514285. Further inquiries can be directed to the corresponding author.
