# Supplementary material for: An ex-vivo model for the biomechanical assessment of cement discoplasty
Source: Front Bioeng Biotechnol. 2022 Sep 2;10:939717. doi: 10.3389/fbioe.2022.939717 (PMC9478659; doi:10.3389/fbioe.2022.939717)
Supplement: Supplementary file 1 [file Image1.PDF]

Supplementary Material

1 General Information

This document contains graphs pertaining compressive testing of functional spinal units. All raw data including microCT images can be found on Zenodo using the following doi: 10.5281/zenodo.6514285

2 Supplementary Figures

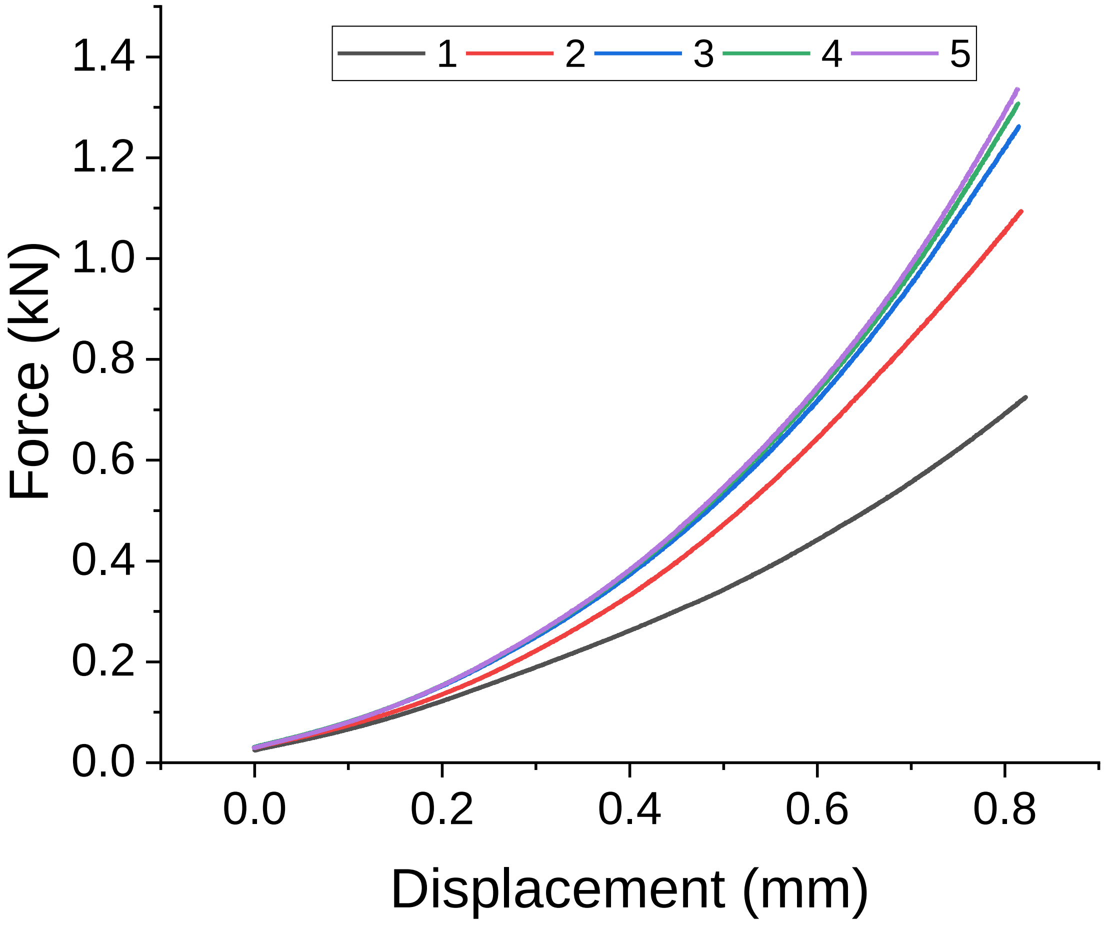

Supplementary Figure 1. Example of an L5-L6 functional spinal unit compression featuring five compression cycles.

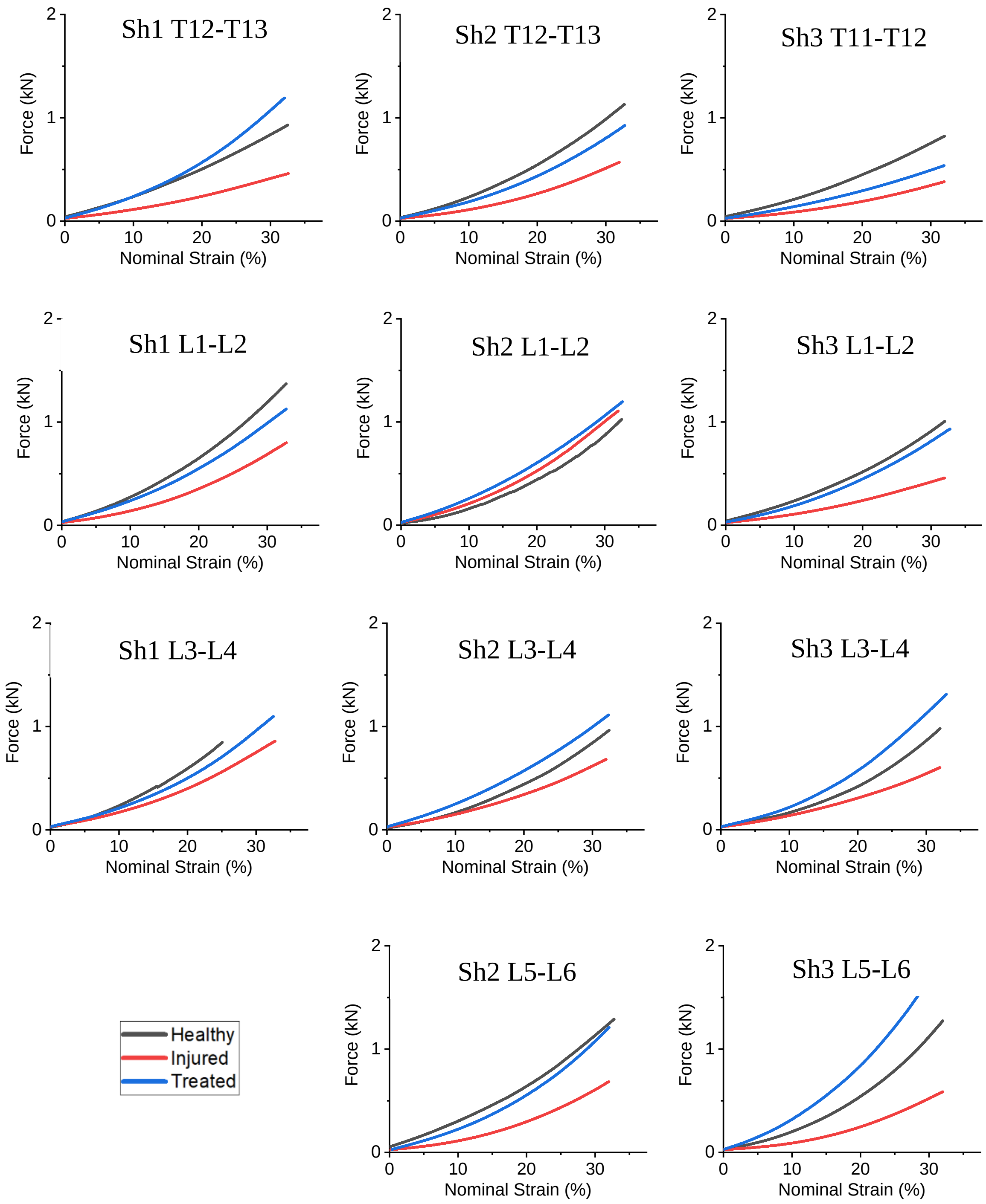

Supplementary Figure 2. Average force vs. displacement curves of the individual spinal segments. The segments were tested 5 times up to 32% nominal strain in compression and the average of the last 3 cycles were used for all health conditions: healthy, injured, and treated. Note that 'Sh1 L3-L4 Healthy' was not tested to 32% due to human error.
